# EnSNAREd by the Sperm Acrosome

**DOI:** 10.1371/journal.pbio.0030352

**Published:** 2005-09-06

Organelles in cells communicate with each other via small membrane-bound vesicles that carry molecular cargo from one part of the cell to another. Just as a cargo ship must know where to dock in order to empty goods at the correct port, vesicles in a cell must dock and empty their contents in the appropriate part of the cell. Studying the regulation of vesicle transport and membrane fusion is a major area of research in cell biology. Though the rules governing vesicle transport and fusion in the sea of cellular organelles have been deciphered in bits and pieces from various cell systems, all the components required for vesicle fusion have not been characterized for any single cell type. In a new study, Gerardo De Blas, Carlos Roggero, Claudia Tomes, and Luis Mayorga have elucidated the molecular mechanics of membrane fusion by studying the single vesicle of the sperm, the acrosome.

Enzymes released from the acrosome facilitate contact between the sperm and egg membranes during fertilization by dissolving the sheath surrounding an egg. For this, the acrosome membrane must fuse with the outer sperm membrane—a process called the acrosome reaction. This reaction happens only once in the lifetime of a sperm. In other systems, proteins involved in membrane fusion must be recycled so they can be reused in the fusion of a subsequent vesicle. But the acrosome reaction is unidirectional and requires no recycling, making it easier to decipher the steps involved.[Fig pbio-0030352-g001]

**Figure pbio-0030352-g001:**
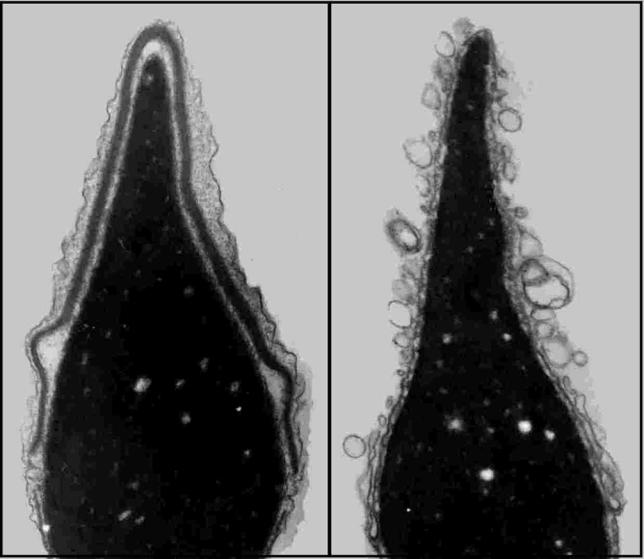
Nonstimulated human sperm (left) and sperm undergoing acrosomal exocytosis (right). Multiple fusion events between the cell membrane and the membrane of the acrosome (the sperm's vesicle) trigger release of acrosomal content and formation of membranebound vesicles, thanks to the action of SNARE complexes

In previous work, Mayorga's group had shown that an increase in the concentration of intracellular calcium activates a molecule called Rab3A, thus initiating the acrosome reaction. The reaction proceeds with the help of various proteins, including NSF, SNAREs, and synaptotagmin VI, as well as calcium release from within the acrosome. Synaptotagmin VI is a calcium-sensitive protein. SNAREs are highly specialized proteins that exist in complexes of three molecules wound together in a helix and are present on both the acrosome and plasma membranes. NSF unwinds these helices so that molecules on opposite membranes can interact. To determine whether the SNARE proteins are in a single or triplet configuration at any given time, the authors used bacterial neurotoxins that can degrade single SNAREs but have no effect against the triplets.

In this study, De Blas and colleagues combine fluorescent techniques, a light-sensitive calcium chelator (which depletes all the calcium in the acrosome), and chemicals that inhibit specific steps in the cascade, to decipher whether each reaction occurs before or after the release of calcium. The researchers show that Rab3A activates NSF, which goes on to untwine helical, neurotoxin-resistant SNARE complexes on the acrosomal and sperm membranes, allowing opposing SNAREs to interact. Once SNAREs on opposite membranes form neurotoxin-sensitive loose complexes, calcium is released from within the acrosome. One protein that is required after the release of calcium is synaptotagmin VI. This protein, the authors suggest, could be responsible for the tight zippering of SNAREs on opposite membranes, converting them into toxin-resistant complexes. As the helix between molecules on opposite membranes becomes tighter, the membranes get pulled closer to each other, enabling membrane fusion.

How calcium activates Rab3A or synaptotagmin VI and how these proteins carry out their roles at the molecular level remain to be elucidated. However, this study elegantly demonstrates the cascade of players involved in the acrosomal membrane fusion reaction, from start to finish, in a single cell—something that had not been shown before. —*Supriya Kumar*

